# Healing Patterns of Periodontal Intrabony Defects Following Allograft and Alloplast Therapy: A Prospective Randomized Clinical Trial

**DOI:** 10.7759/cureus.103311

**Published:** 2026-02-09

**Authors:** Shubham Sharma, Nitin Tomar, Sameer Ahmed, Mayur Kaushik

**Affiliations:** 1 Department of Periodontology and Oral Implantology, Subharti Dental College and Hospital, Swami Vivekananda Subharti University, Meerut, IND; 2 Department of Periodontology, Subharti Dental College and Hospital, Swami Vivekananda Subharti University, Meerut, IND

**Keywords:** biphasic calcium phosphate, bone graft materials, freeze-dried bone allograft, intrabony periodontal defects, periodontal regeneration

## Abstract

Background: Vertical periodontal bone defects pose a significant challenge in regenerative therapy. This study compared the clinical and radiographic outcomes of freeze-dried bone allograft (FDBA) and biphasic calcium phosphate (adbone®BCP) in patients with chronic periodontitis.

Methods: Twenty systemically healthy patients were randomized into two groups to receive either FDBA or AdboneBCP following open flap debridement. Clinical parameters, pocket probing depth (PPD) and relative attachment level (RAL), and radiographic bone fill (RBF) were assessed at baseline, three months, and six months postoperatively.

Results: Both graft materials led to significant improvements over six months. Mean PPD reduction was 4.2 mm with FDBA and 3.9 mm with AdboneBCP; mean RAL gain was 3.8 mm and 3.5 mm, respectively. Radiographic bone fill averaged 62% for FDBA and 58% for AdboneBCP. Differences between groups were not statistically significant. No adverse events were reported.

Conclusions: AdboneBCP is a safe, effective, and predictable synthetic alternative to FDBA for regenerating vertical periodontal defects. Its comparable clinical and radiographic outcomes support its use in scenarios where allograft availability is limited.

## Introduction

Each periodontal defect tells the silent story of tissue breakdown, while every graft embodies the potential for regeneration. Periodontal disease is a chronic, progressive immune-mediated disorder of the periodontium that compromises the integrity of its supporting structures, including the gingiva, periodontal ligament, cementum, and alveolar bone. The fundamental aim of periodontal therapy extends beyond halting disease progression to achieving true regeneration of these lost tissues. Among the various regenerative modalities, bone grafting continues to serve as a cornerstone approach [1].

Freeze-dried bone allograft (FDBA) has long been recognized as a reliable material in periodontal regeneration, attributed to its excellent biocompatibility and osteoconductive potential. It provides a demineralized scaffold that facilitates cellular adhesion with bone formation. Earlier studies demonstrated its efficacy in bone regeneration enhancement and an increase in the level of clinical attachment within periodontal intrabony defects, proving its regenerative potential [2]. On the other hand, various bone replacement grafts, including allografts and synthetic calcium phosphate-based materials, have demonstrated predictable regenerative outcomes in periodontal intrabony defects [3,4].

The biphasic calcium phosphate (BCP) graft, marketed as adbone®BCP (Medbone Biomaterials, Sintra, Portugal), comprising 75% hydroxyapatite (HA) and 25% β-tricalcium phosphate, is a synthetic substitute designed to closely replicate the mineral composition of native bone while offering predictable resorption characteristics [5]. Comparative evaluation between BCP and demineralized FDBA (DFDBA) has shown comparable clinical and radiographic outcomes, providing validation for the effectiveness of lab-manufactured substitutes such as adboneBCP in periodontal regeneration [6].

AdboneBCP has excellent handling characteristics and biocompatibility with predictable outcomes. This study aimed to juxtapose FDBA and adboneBCP in the treatment of vertical bone defects associated with periodontitis by systematically collating patient-based and radiomorphometric parameters over a six-month follow-up period. 

## Materials and methods

This was a single-blinded, randomized, prospective, concurrent-group clinical trial, conducted at Subharti Dental College and Hospital, Swami Vivekanand Subharti University, Meerut, Uttar Pradesh, India, from February 2025 to October 2025, in accordance with the Consolidated Standards of Reporting Trials (CONSORT) guidelines [7]. Ethical approval was obtained from the University Ethics Committee (Medical), Swami Vivekanand Subharti University (reference number: SMC/UECM/2023/640/296). The trial was registered with the Clinical Trials Registry of India (CTRI) (Registration No.: CTRI/2025/01/078883). Written informed consent was obtained from all participants prior to enrollment.

Inclusion and exclusion criteria

Patients were included in the study if they exhibited a probing pocket depth (PPD) greater than 5 mm, the presence of a vertical intrabony periodontal defect confirmed by both clinical and radiographic examination, and vitality of the involved tooth. Patients were excluded if they had any systemic conditions known to affect periodontal healing, were pregnant or lactating, had a history of smoking or tobacco use, or had undergone any form of periodontal therapy within the preceding six months.

Sample size calculation

**Figure 1:**
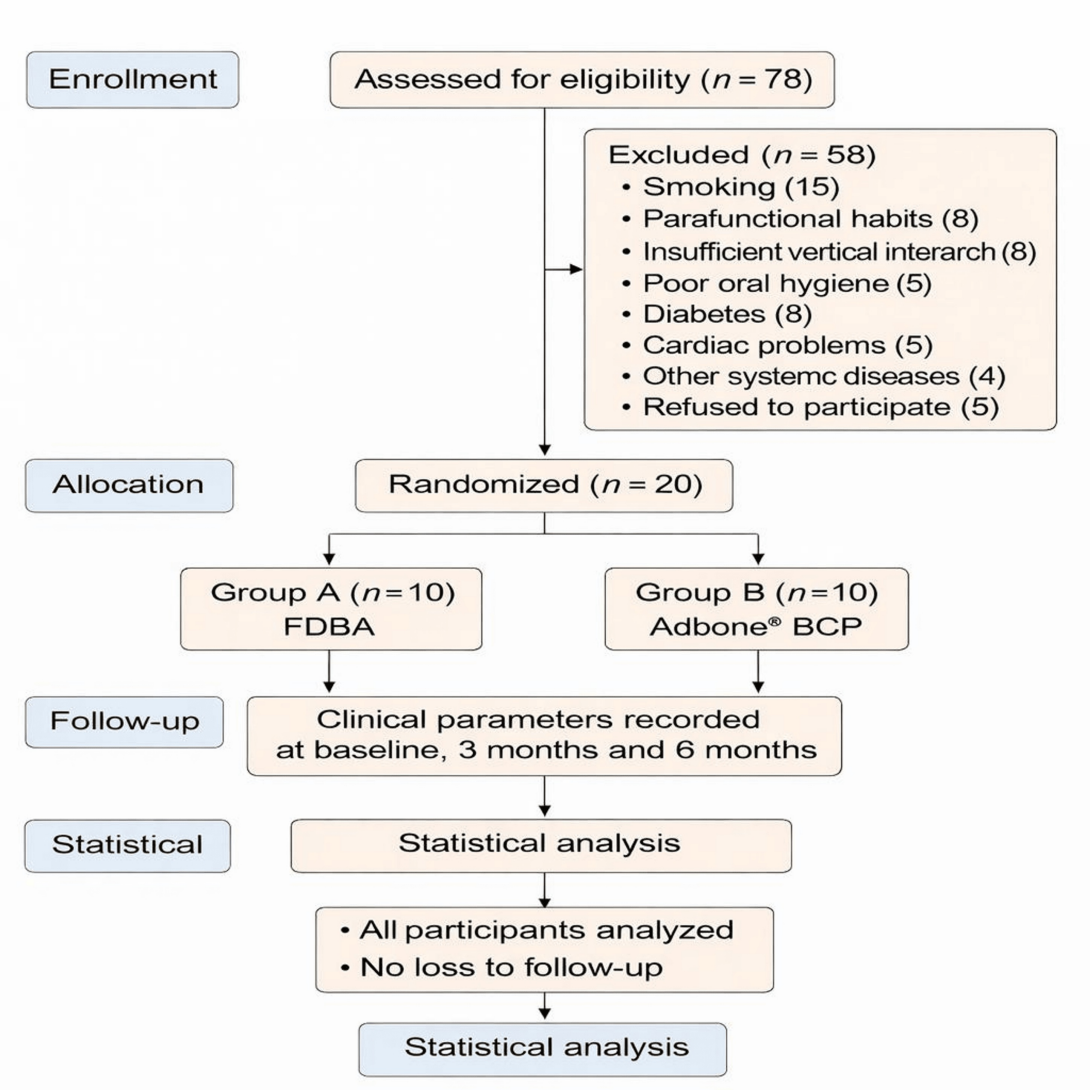
Patient allotment FDBA: freeze-dried bone allograft; BCP: biphasic calcium phosphate

A total of 78 patients were screened, of which 20 systemically healthy individuals (aged 25-55 years) diagnosed with chronic periodontitis and presenting with vertical intrabony periodontal defects were selected for inclusion. The sample size was calculated using G*Power software (version 3.0.10), based on the primary outcome variable of PPD reduction. An effect size of 1.04, derived from a previously published study [8], was used for the sample size estimation. With a power of 80% and a significance level of 0.05, the required sample size was estimated to be 10 sites per group. Each participant contributed a single intrabony defect site, primarily located in posterior teeth, to avoid clustering effects (Figure 1).

Randomization, allocation concealment, and blinding 

Participants who fulfilled the eligibility criteria and provided informed consent were allocated to the Control (FDBA) or Test (adboneBCP) groups using a simple randomization method. A computer-generated random sequence was created prior to participant allocation to ensure an equal probability of assignment to either group.

Allocation concealment was ensured using opaque, sealed, sequentially numbered envelopes containing the group assignments. These envelopes were opened only after participant enrollment and completion of baseline recordings, thereby preventing foreknowledge of treatment allocation and minimizing selection bias.

This study followed a single-blinded design in accordance with CONSORT 2025 guidelines. Patients were blinded to the type of graft material used. Due to the nature of the surgical procedure and the distinct handling characteristics of the graft materials, blinding of the operator was not feasible. To maintain procedural consistency, all surgical interventions were performed by a single operator (the principal investigator).

Clinical measurements

All clinical measurements were recorded by a single calibrated examiner. Examiner calibration was performed prior to the initiation of the study by repeating measurements at selected sites, and intra-examiner reliability was assessed. The intraclass correlation coefficient (ICC) demonstrated good to excellent reliability for clinical parameters, including Plaque Index (PI), Gingival Index (GI), PPD, relative attachment level (RAL), and radiographic bone fill (RBF). This confirmed the consistency and reproducibility of clinical and radiographic measurements throughout the study period.

Study objectives and outcome measures

The study objectives were clearly defined and categorized into primary and secondary outcome variables. The primary outcome variables included a reduction in PPD and RBF. Secondary outcome variables comprised changes in RAL, PI, and GI. The study was designed to compare the clinical and radiographic performance of FDBA and adboneBCP in the treatment of intrabony periodontal defects.

Phase I therapy and baseline assessment

Prior to surgical intervention, all participants underwent comprehensive Phase I periodontal therapy, including scaling and root planing. Baseline clinical parameters were recorded following completion of initial therapy and included PI as described by Silness and Löe (1964) [9], GI as described by Löe and Silness (1963) [10], PPD, and RAL. All clinical measurements were obtained using a UNC (University of North Carolina)-15 periodontal probe by a single calibrated examiner to ensure measurement accuracy and reproducibility.

Surgical procedure

**Figure 2:**
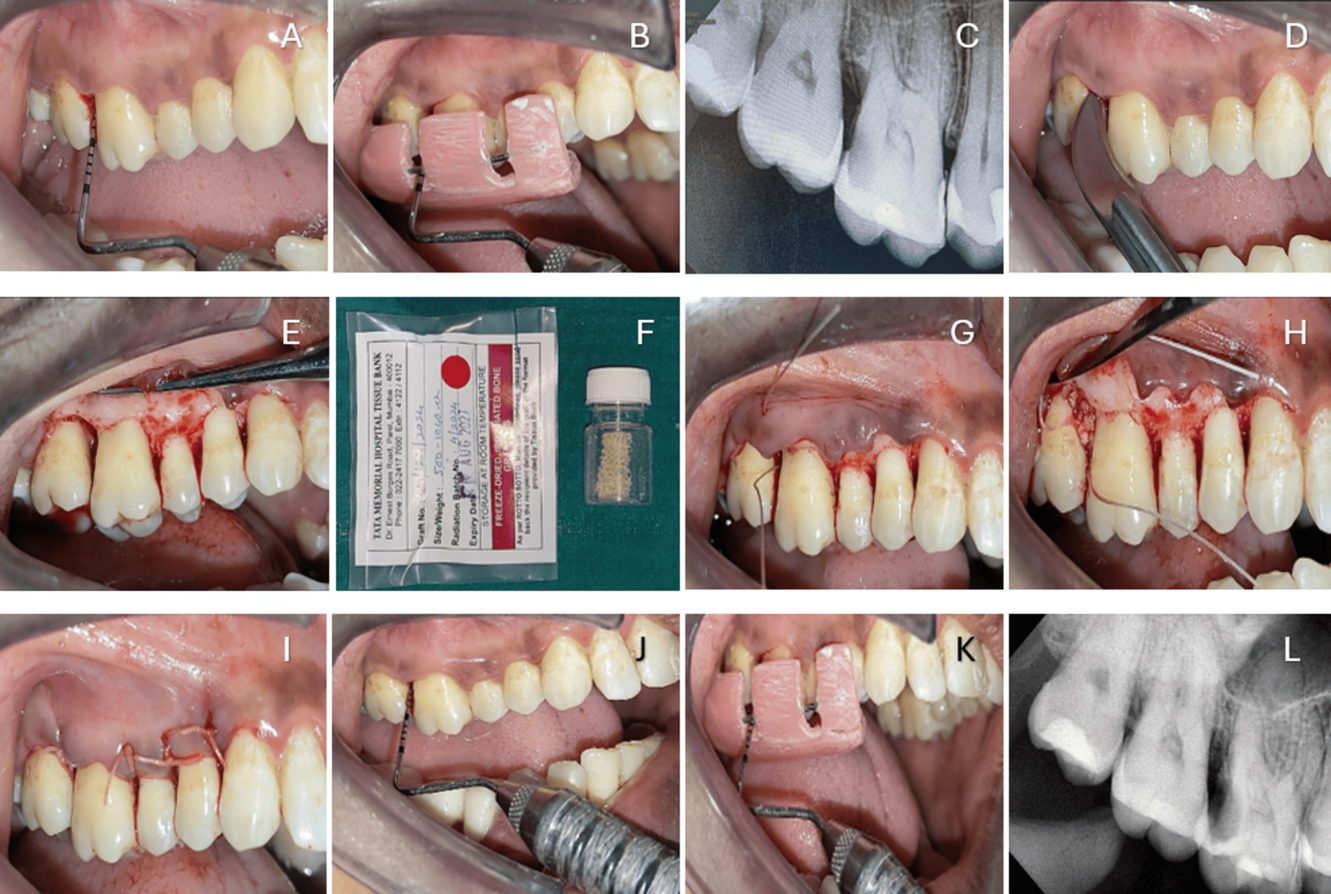
(A) PPD at baseline; (B) RAL at baseline; (C) RVG at baseline; (D) crevicular incision; (E) defect area debrided; (F) FDBA bone graft; (G) pre-suturing; (H) graft condensed; (I) sutures given; (J) PPD at six months; (K) RAL at six months; (L) RVG at six months PPD: probing pocket depth; RAL: relative attachment level; RVG: radiovisiography, FDBA: freeze-dried bone allograft

**Figure 3:**
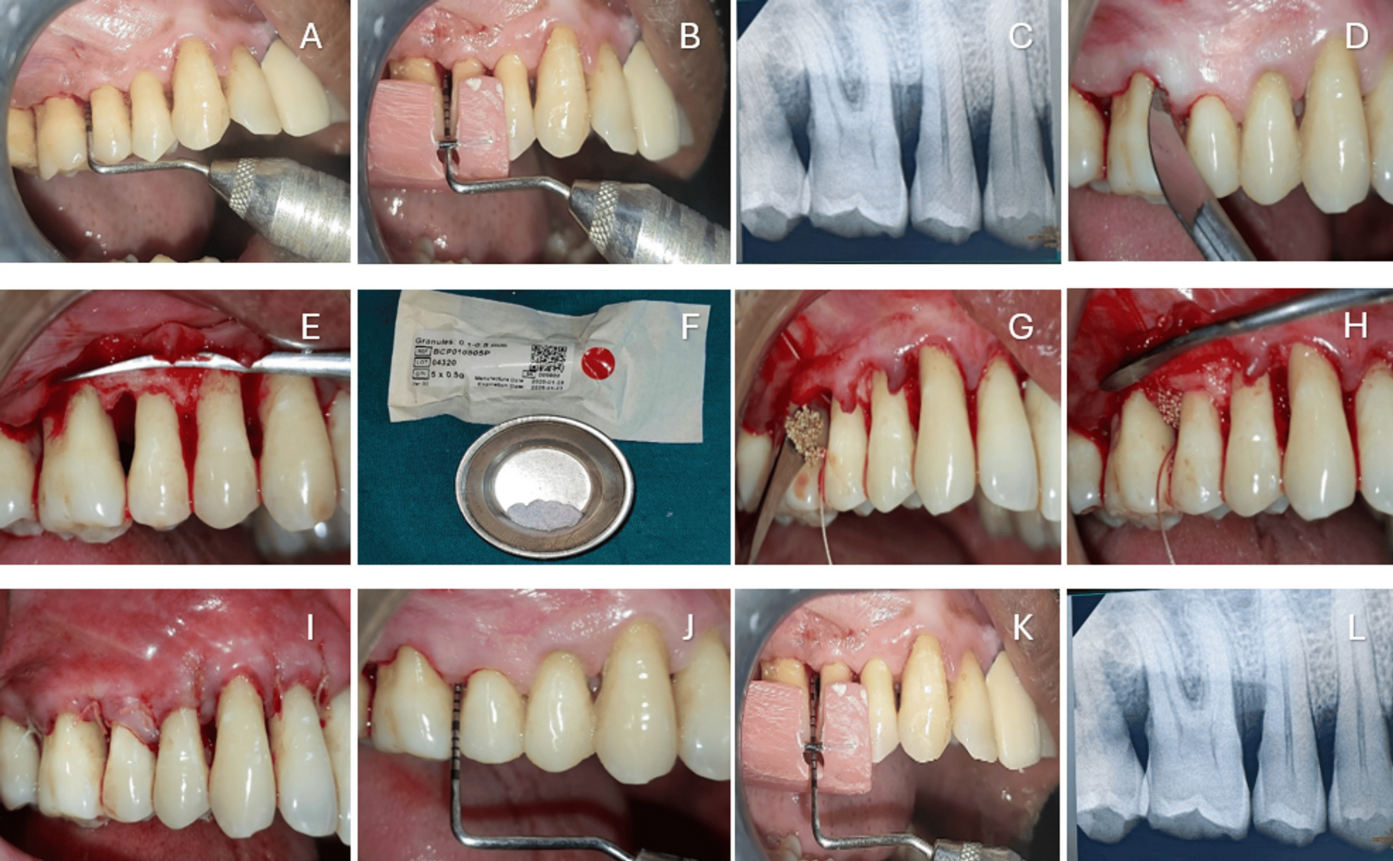
(A) PPD at baseline; (B) RAL at baseline, (C) RVG at baseline; (D) crevicular incision; (E) defect area debrided; (F) Adbone®BCP bone graft; (G) pre-suturing; (H) graft condensed; (I) sutures given; (J) PPD at six months; (K) RAL at six months; (L) RVG at six months PPD: probing pocket depth; RAL: relative attachment level; RVG: radiovisiography

All surgical procedures were performed by a single operator (principal investigator) under local anesthesia. Following reflection of a full-thickness mucoperiosteal flap, meticulous debridement and root planing of the intrabony defect were carried out. In Group A, FDBA obtained from the Tissue Bank of Tata Memorial Hospital, Mumbai, Maharashtra, India, was placed in the defect between teeth 16 and 17 (Figure 2). In Group B, adboneBCP was placed in the defect between teeth 15 and 16 (Figure 3). The quantity of graft material used was sufficient to completely fill the defect without overpacking and was determined based on the size and morphology of the defect. No barrier membrane or additional scaffold was employed. Following graft placement, the mucoperiosteal flaps were repositioned to achieve primary closure and secured using resorbable polyglycolic acid (PGA)-polylactic acid (PLA) sutures in a figure-of-eight configuration to enhance graft stability. A non-eugenol periodontal dressing was applied to protect the surgical site.

Postoperative care

Postoperative instructions were provided to all patients. The prescribed medication regimen included a 2% povidone-iodine mouth rinse for the first two days owing to its hemostyptic and antiseptic properties, followed by 0.12% chlorhexidine gluconate mouth rinse twice daily for two weeks. Systemic antibiotic therapy consisting of amoxicillin 500 mg with clavulanic acid 125 mg (Moxikind-CV) was prescribed, along with analgesic medication comprising aceclofenac 100 mg, paracetamol 325 mg, and serratiopeptidase 15 mg (Zerodol-SP). Mechanical plaque control at the surgical site was avoided during the initial healing period. Sutures and the periodontal dressing were removed after 7-10 days, and healing was monitored during subsequent follow-up visits.

Clinical and radiographic evaluation

Clinical parameters (PPD and RAL) were recorded at baseline, three months, and six months using a UNC-15 probe. RAL measurements were obtained using a customized acrylic stent as a reference point to ensure reproducibility. PPD was measured from the marginal gingiva to the deepest probing depth, while RAL was measured from the stent reference point to the base of the defect. Standardized intraoral periapical radiographs were obtained using the paralleling technique at baseline and six months.

RBF assessment

Radiomorphometric analysis was performed using digital image analysis software. The cemento-enamel junction (CEJ) (Point A) was used as the primary reference landmark; in cases where the CEJ was obscured by restorative treatment, the restoration margin was considered. The alveolar crest (Point B) and the most coronal point of the bony defect (Point C) were identified, and the bone defect area (BDA) was calculated using the formula described by Eickholz et al. [11], BDA = ½ x AC x AB.

**Figure 4:**
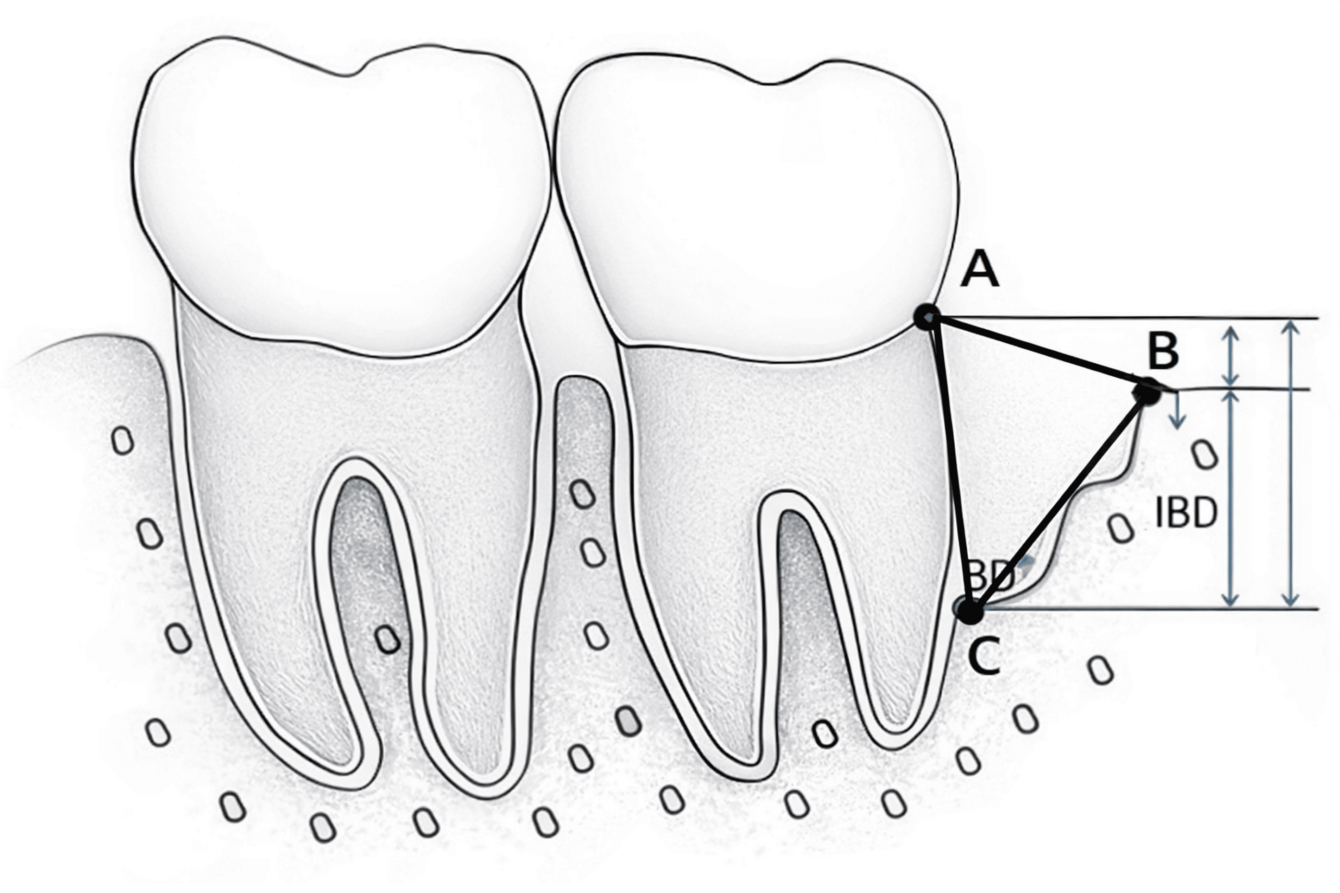
Diagrammatic representation to calculate the area of the defect A: cemento-enamel junction; B: alveolar crest; C: most coronal point of the bony defect Image adapted from: Lee et al., 2012 [12]; used under the terms of the Creative Commons Attribution Non-Commercial License CC BY-NC 3.0 Attribution-NonCommercial 3.0 Unported deed (http://creativecommons.org/licenses/by-nc/3.0/)

A diagrammatic representation illustrating the calculation of the defect area is shown in Figure 4.

RBF percentage was calculated as: RBF (%) = $\frac{(BDA\,\operatorname{at}\,baseline - BDA\,\operatorname{at}\,6\,months)\times 100}{BDA\,\operatorname{at}\,baseline}$

Measurements were calibrated digitally using the distance from CEJ (Point A) to the alveolar crest (Point B) and CEJ (Point A) to bony defect (Point C) at baseline and six months.

Follow-up duration and data presentation

Intermediate evaluations were conducted at three months; however, these did not show statistically significant differences compared to baseline. Therefore, to maintain focus on clinically relevant outcomes and avoid unnecessary manuscript length, only baseline and six-month data were presented, with six months designated as the primary endpoint.

Statistical analysis

The collected data were statistically evaluated using IBM SPSS Statistics for Windows, version 25.0 (IBM Corp., Armonk, New York, United States). Descriptive analyses, comprising mean and standard deviation (SD) values, were computed for all clinical and radiographic parameters at each follow-up interval.

Data were assessed for normality using the Shapiro-Wilk test. PI values did not follow a normal distribution, whereas GI, PPD, RAL, and RBF were normally distributed. Within-group comparisons between baseline and follow-up time points (three and six months) were conducted using the paired t-test for normally distributed variables and the Wilcoxon signed-rank test for non-normally distributed variables. Intergroup comparisons between the Control (FDBA) and Test (AdboneBCP) groups were performed using the independent (unpaired) t-test for normally distributed data and the Mann-Whitney U test for non-normally distributed data. A p-value < 0.05 was considered statistically significant for all analyses.

## Results

The test and control groups demonstrated baseline comparability (Table 1), but there were substantial improvements in all patient-based parameters, PI, GI, PPD, and RAL, from baseline to six months (p < 0.001) (Table 2), while radiographic analysis revealed a mean percentage bone fill of 29.58 ± 21.23% in FDBA and 26.09 ± 12.86% in AdboneBCP at the six-month evaluation (Table 3). At the six-month follow-up, FDBA showed a slightly greater reduction in PPD and RAL, which corresponds to a gain in periodontal attachment and a slightly higher mean radiographic bone gain juxtaposed to AdboneBCP. However, this intergroup difference did not attain statistical significance. These findings indicate that both grafting materials exhibit comparable regenerative potential in the management of vertical bone defects associated with periodontitis.

**Table 1:** Baseline comparison of clinical parameters between Group A (FDBA) and Group B (Adbone®BCP) Baseline intergroup comparisons were performed using the independent t-test for normally distributed variables (GI, PPD, RAL) and the Mann–Whitney U test for non-normally distributed variables (PI). FDBA: freeze-dried bone allograft; BCP: biphasic calcium phosphate

Clinical Parameters	Group A - Baseline (n=10), mean ± SD	Group B - Baseline (n=10), mean ± SD
Plaque Index (PI)	1.53 ± 0.24	1.45 ± 0.32
Gingival Index (GI)	1.18 ± 0.23	1.17 ± 0.30
Probing Pocket Depth (PPD) (mm)	7.2 ± 1.31	6.4 ± 1.17
Relative Attachment Level (RAL) (mm)	9.9 ± 1.52	8.7 ± 0.97

**Table 2:** Comparison of clinical parameters at baseline and six months between Group A (FDBA) and Group B (Adbone®BCP) Intragroup comparisons between baseline and six months were performed using the paired t-test for normally distributed variables (GI, PPD, RAL) and the Wilcoxon signed-rank test for non-normally distributed variables (PI). A p-value < 0.05 was considered statistically significant. FDBA: freeze-dried bone allograft; BCP: biphasic calcium phosphate

Clinical Parameters	Group A – Baseline (n=10), mean ± SD	Group A – 6 Months (n=10), mean ± SD	p-value	Group B – Baseline (n=10), mean ± SD	Group B – 6 Months (n=10), mean ± SD	p-value
Plaque Index (PI)	1.53 ± 0.24	1.21 ± 0.16	0.012	1.45 ± 0.32	1.20 ± 0.28	0.015
Gingival Index (GI)	1.18 ± 0.23	1.10 ± 0.18	0.042	1.17 ± 0.30	1.05 ± 0.51	0.048
Pocket Probing Depth (PPD) (mm)	7.2 ± 1.31	4.4 ± 1.17	0.001	6.4 ± 1.17	3.2 ± 0.79	0.001
Relative Attachment Level (RAL) (mm)	9.9 ± 1.52	6.2 ± 1.28	0.001	8.7 ± 0.97	5.9 ± 1.44	0.001

**Table 3:** Radiographic bone fill (RBF) and radiographic bone fill percentage (RBF%) in Group A (FDBA) and Group B (Adbone®BCP) Intergroup comparison of radiographic parameters (RBF and defect area reduction) was performed using the independent t-test. FDBA: freeze-dried bone allograft; BCP: biphasic calcium phosphate

Groups	Mean Defect Area Reduction (mm²)	Mean RBF (mm²)	RBF%, mean ± SD
Group A	2.65	2.68	29.58 ± 21.23
Group B	2.68	2.66	26.09 ± 12.86

## Discussion

In the landscape of periodontal healing, it is not the material alone but its harmony with biology that defines success. This study assessed and compared the patient-based and radiomorphometric outcomes of FDBA and AdboneBCP in the management of vertical bone defects associated with periodontitis. Both groups showed substantial improvements in patient-based measures and RBF during the six-month follow-up period.

The reduction in PPD and the gain in RAL observed in both groups highlight the regenerative potential of the grafting materials, but FDBA exhibited marginally greater patient-based and radiomorphometric improvements compared to AdboneBCP. These outcomes are in accordance with previous studies exhibiting osteoconductive and regenerative properties of FDBA by providing a scaffold for cellular attachment and new bone formation. The observed reductions in probing depth and gains in relative attachment level are consistent with previous reports demonstrating favorable clinical outcomes following regenerative therapy of intrabony defects using bone graft materials [3,13].

At the six-month evaluation, both treatment groups exhibited substantial improvements across all clinical parameters, including PPD and RAL. In the FDBA group, the mean PPD decreased from 7.2 ± 1.31 mm to 4.4 ± 1.17 mm, accompanied by a RAL decrease from 9.9 ± 1.52 mm to 6.2 ± 1.28 mm. AdboneBCP showed a decrease in PPD from 6.4 ± 1.17 mm to 3.2 ± 0.79 mm and in RAL from 8.7 ± 0.97 mm to 5.9 ± 1.44 mm, which corresponds to a gain in periodontal attachment (Table 1). These clinical results align with previous studies comparing AdboneBCP to DFDBA, which reported similar clinical and radiographic outcomes, supporting the efficacy of synthetic alternatives such as AdboneBCP in periodontal regeneration [14]. Likewise, comparable clinical responses between BCP and DFDBA have been observed in the management of vertical osseous defects [15].

**Figure 5:**
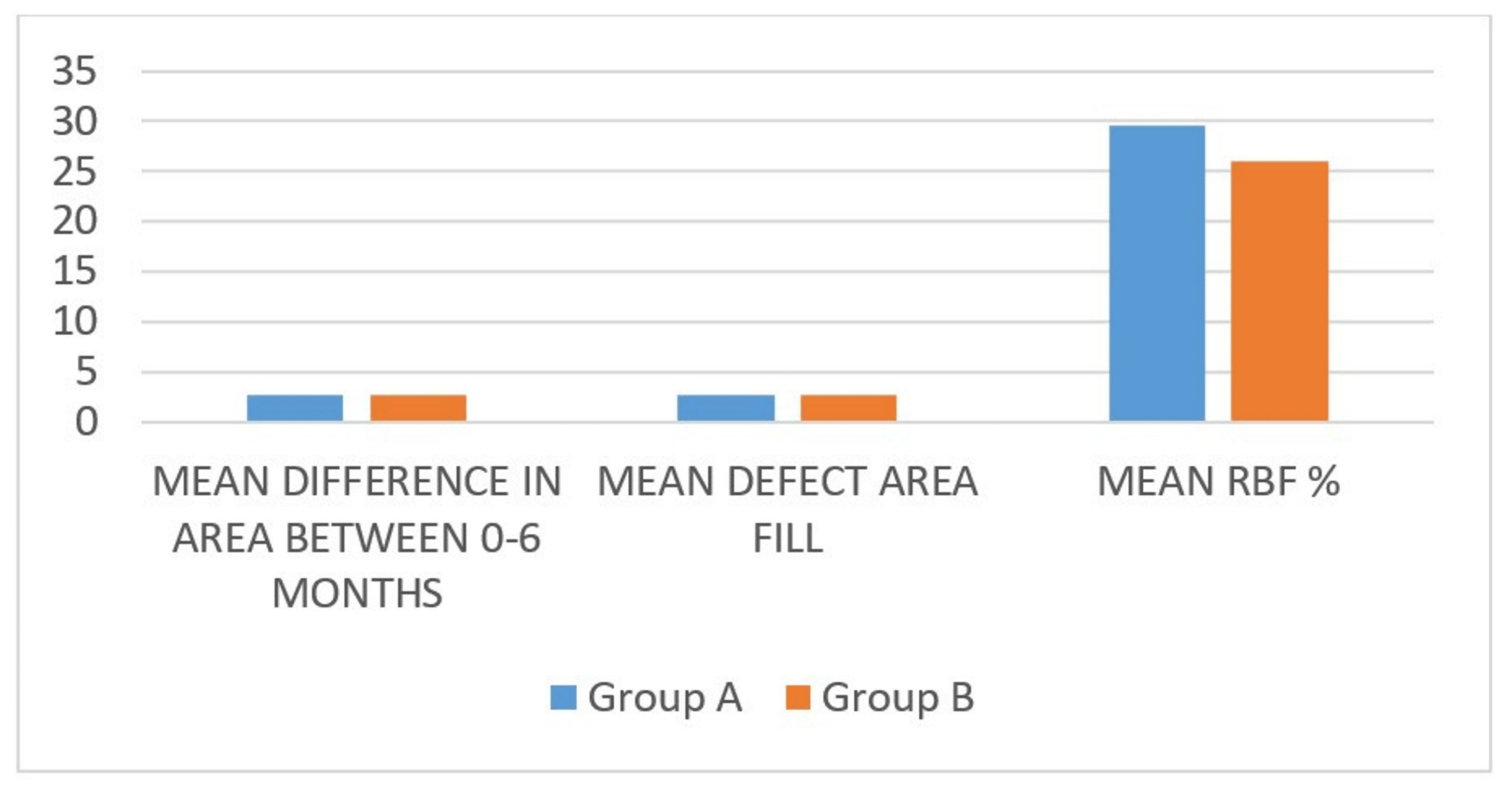
The bar diagram of mean difference in area, RBF, and RBF% in Group A (FDBA) and Group B (Adbone®BCP) RBF: radiographic bone fill; FDBA: freeze-dried bone allograft; BCP: biphasic calcium phosphate

Radiographic analysis revealed a mean bone fill of 29.58 ± 21.23% in FDBA and 26.09 ± 012.86% in AdboneBCP, with RBF values of 2.68 mm² and 2.66 mm², respectively (Table 2). These findings (Figure 5) are consistent with previous studies highlighting the dependable regenerative capability of FDBA, attributed to its osteoconductive scaffold that promotes cellular proliferation and bone formation[16].

Nevertheless, the FDBA group showed slightly greater improvements in all assessed parameters, PPD, RAL, and RBF. Overall, the intergroup differences were not statistically significant (p > 0.05), indicating that both groups achieved comparable clinical outcomes.

AdboneBCP, consisting of HA and BCP, offers a biocompatible and resorbable scaffold that facilitates new bone formation. The positive outcomes observed in this study align with the findings of MacBeth et al. [17], demonstrating that BCP effectively maintains ridge volume and promotes hard tissue regeneration. Additionally, histological studies have shown a favorable tissue response to BCP in human extraction sockets, supporting its clinical use [18].

These findings are consistent with earlier studies comparing allogenic grafts and autografts in intrabony defects, both of which showed significant improvements without significant differences between groups [8]. Furthermore, clinical evidence supports the effective use of FDBA combined with platelet-rich fibrin (PRF) to enhance periodontal tissue regeneration, highlighting the role of allografts in promoting tissue healing [19]. Similar observations have been reported in comparative studies evaluating adjunctive biologic modifiers in combination with bone grafts [20]. Conversely, a comparative study reported that the adjunctive use of platelet-rich fibrin with DFDBA did not confer additional regenerative benefit over DFDBA alone in the treatment of intrabony periodontal defects [21].

No adverse events or complications occurred during the follow-up period, confirming the clinical safety and biocompatibility of both graft materials. Various studies have indicated that while FDBA enhances initial bone regeneration, BCP contributes to structural stability and increased mineralization in the later stages of healing [22]. Previous studies have indicated that regenerative periodontal therapy for intrabony defects may provide sustained clinical improvements and favourable tooth retention over time, highlighting its potential role in managing severely compromised teeth [23]. These observations are in conjunction with the current outcomes and emphasize the significance of choosing graft materials based on clinical priorities as early healing, scaffold stability, and long-term bone maturation.

Clinically, the findings indicate that both FDBA and BCP are effective for the management of vertical intrabony defects, demonstrating comparable improvements in probing depth reduction, clinical attachment gain, and radiographic bone fill. The utilisation of synthetic graft materials, such as AdboneBCP, may represent a viable alternative to allografts, particularly in contexts where availability, cost, or patient preference constrain the use of allogeneic material.

The limitations and future directions of the present study include a relatively short follow-up period of six months and a limited sample size, which may restrict assessment of long-term stability and generalizability of the findings. In addition, reliance on two-dimensional radiographic evaluation may not fully represent volumetric bone changes. Future investigations should incorporate longer follow-up durations, larger sample sizes, three-dimensional imaging modalities such as cone-beam computed tomography, and histological assessment. The adjunctive use of biologic modifiers or growth factors may also be explored to further enhance periodontal regenerative outcomes.

## Conclusions

This study elucidated that both FDBA and AdboneBCP facilitated substantive and clinically relevant enhancements in both clinical and radiographic outcomes in the management of periodontal intrabony defects. Although FDBA demonstrated a modest predilection toward superior bone fill and attachment level gains, the observed intergroup differences were not statistically discernible, underscoring the comparable regenerative potential of both biomaterials.
